# Recurrent Kawasaki Disease Presenting to Dentists: “Think Beyond Dentition”

**DOI:** 10.5005/jp-journals-10005-1571

**Published:** 2018

**Authors:** Leena Verma, Sidhi Passi, Gagandeep Kaur, Jyoti Gupta, Mitul Joshi

**Affiliations:** 1,2 Department of Pedodontics, Dr Harvansh Singh Judge Institute of Dental Sciences, Panjab University, Chandigarh, India; 3,5 Department of Pedodontics, College of Dental Sciences, Amargarh, Bhavnagar, Gujarat, India; 4 Department of Public Health Dentistry, Dr Harvansh Singh Judge Institute of Dental Sciences, Panjab University, Chandigarh, India

**Keywords:** Kawasaki disease, Orofacial features, Recurrent

## Abstract

**Aim:**

To create awareness of Kawasaki disease in the dental community as it is a rare disease and some cases might go unnoticed due to lack of knowledge of the treating dentist. Thus proper knowledge helps in the timely diagnosis of the disease and decrease the mortality rate in these patients. Children who show the oral features of this disease should be treated seriously by the dentist.

**Background:**

Kawasaki disease is a rare acute systemic vasculitis disorder reported in the dental literature. Clinical features include high fever, redness of palms and soles of the feet, conjunctivitis, oropharyngeal mucositis and lymphadenopathy. The cardiac involvement in the form of myocarditis, aneurysms, pericarditis is also seen and is caused by inflammation of vessels of the heart.

**Case description:**

Here we present a rare case of an 8-year-old girl who presented to the department of Pediatric Dentistry with the chief complaint of recurrent painless swelling of the lower lip. This rare presentation of lower lip swelling has not been cited in the oral manifestation of Kawasaki disease before.

**Clinical significance:**

The disease has high mortality and morbidity rate if not treated early, and hence an early diagnosis and treatment are important in managing this condition. The oral findings are a characteristic feature of this serious disease, hence, many cases might first report to the dental clinician only. Dentists should always remain alert in handling patients having a history of Kawasaki disease because of the possibility of recurrence of the disease. As these patients have valvular heart defects, they might require prophylactic antibiotic treatment before the needed dental procedure.

**Conclusion:**

Despite this, there seems to be less aware of this disease among the dentist, hence this condition goes unnoticed leading to few citations of this disease in the dental literature.

**How to cite this article:**

Verma L, Passi S, Kaur G, Gupta J, Joshi M. Recurrent Kawasaki Disease Presenting to Dentists: “Think Beyond Dentition”. Int J Clin Pediatr Dent, 2018;11(6):532-535

## BACKGROUND

Kawasaki disease (KD) is a rare disorder of children with an annual incidence of 6.2/100,000 per children. It is usually seen more in boys and is characterized by fever for more than 5 days, rash, swelling in hands and feet, redness and irritation in the eye, lymph glands swelling in the neck, and erythema of the lips, oral mucosa, and throat.^1,2^ It is named after Dr. Tomisaku Kawasaki, a Japanese pediatrician who said that this disease almost always affects children who are under 5 years age.^3^ The incidence of the disease is higher in Japan than in any other country.^4,5^

As proven by epidemiological studies and clinical presentation, the disease is infective in origin.^6^ So no specific etiological agent could be identified so far; therefore, the infection is a triggering factor for the disease in genetically susceptible subjects.^7,8^

The diagnosis of the disease can be done by the following features: persistent fever which lasts at least 5 days and does not disappear with the usual antipyretic drugs; polymorphous rash; conjunctival congestion; oropharyngeal mucositis (erythematous and cracked lips, strawberry tongue, pharyngeal erythema), swelling and peeling on upper and lower limbs, and laterocervical lymphadenitis.^9^ These clinical features can be associated with irritability, diarrhea, hepatitis, hydrops of gallbladder, urethritis, otitis media, meningism, and arthritis.^9–11^

The disease usually presents with an average time period of 6–8 weeks and occurs in 3 stages. The first stage is the acute febrile stage which lasts for 1–2 weeks followed by subacute stage which is of approximately 25 days and is characterized by desquamation, arthralgia, and increased platelets count. In the last phase, i.e. convalescent phase, clinical signs disappear and ESR return normal.^12^

Here we present a rare case of an 8-year-old girl who presented to Department of pediatric dentistry with painless swelling of lower lip which has very rarely been reported in the oral manifestation of this disease, thus making this case report a novel presentation of Kawasaki disease. The early diagnosis of recurrent Kawasaki disease by the dentist led to appropriate management of the patient and prevented morbidity and mortality.

## CASE DESCRIPTION

**Fig. 1:**
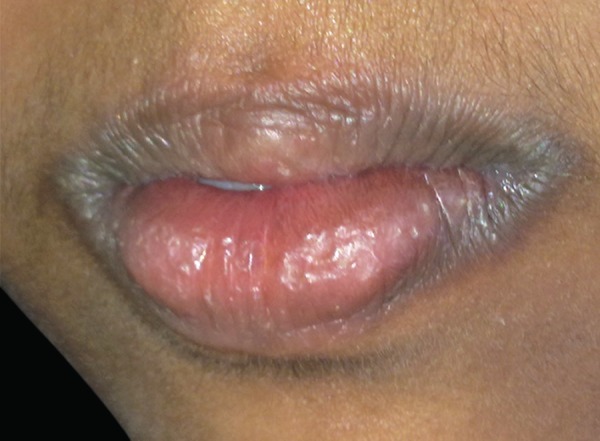
Extraoral picture showing lower lip swelling and drying and cracking of lips

An 8-year-old girl reported to pedodontic clinics with mild pyrexia, lethargicness lower lip swelling, and a sore tongue. The lymph nodes were significantly enlarged. On oral examination, lips were found to be dry, cracked, red, and localized swelling was seen of the lower lip (Fig. 1). This swelling was accompanied by itching and subsided on its own. This painless swelling of lower lip has very rarely been reported in the oral manifestation of this disease, thus making this case report a novel presentation of Kawasaki disease. The patient first reported lower lip swelling and after few days strawberry tongue was seen. Her past medical history revealed that the child had developed Kawasaki disease at the age of 4 years for which she was hospitalized for uncontrolled fever.

**Fig. 2:**
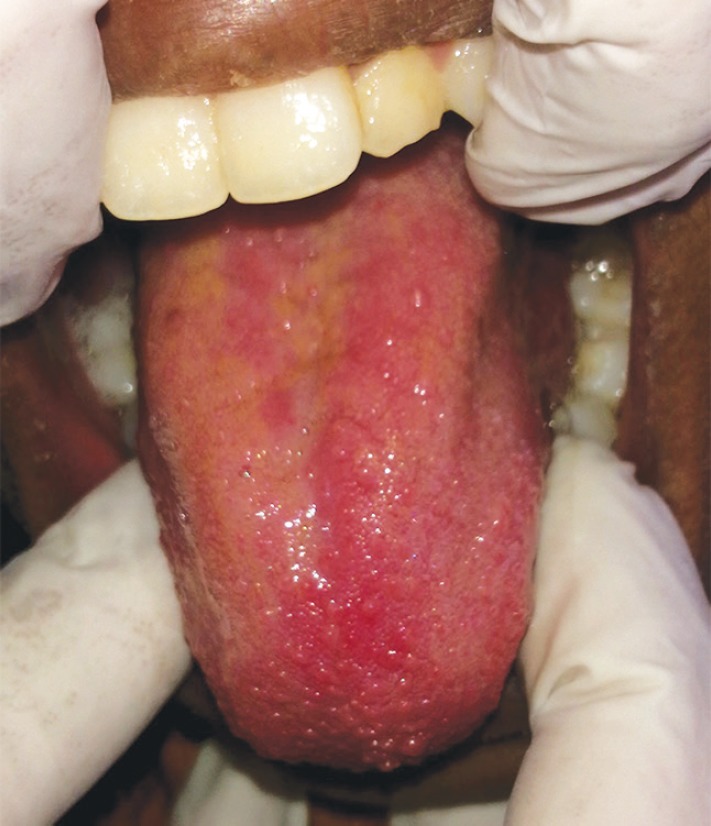
Intraoral picture showing from the presence of a brightly erythematous tongue called as ‘strawberry’ tongue along with tongue tie

**Fig. 3:**
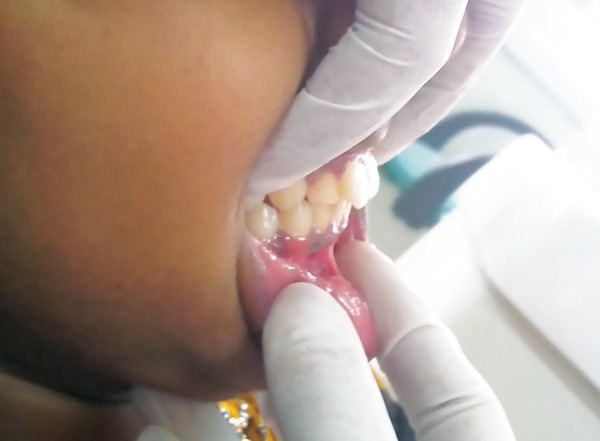
Intraoral picture showing the presence of protruded maxillary teeth

**Fig. 4:**
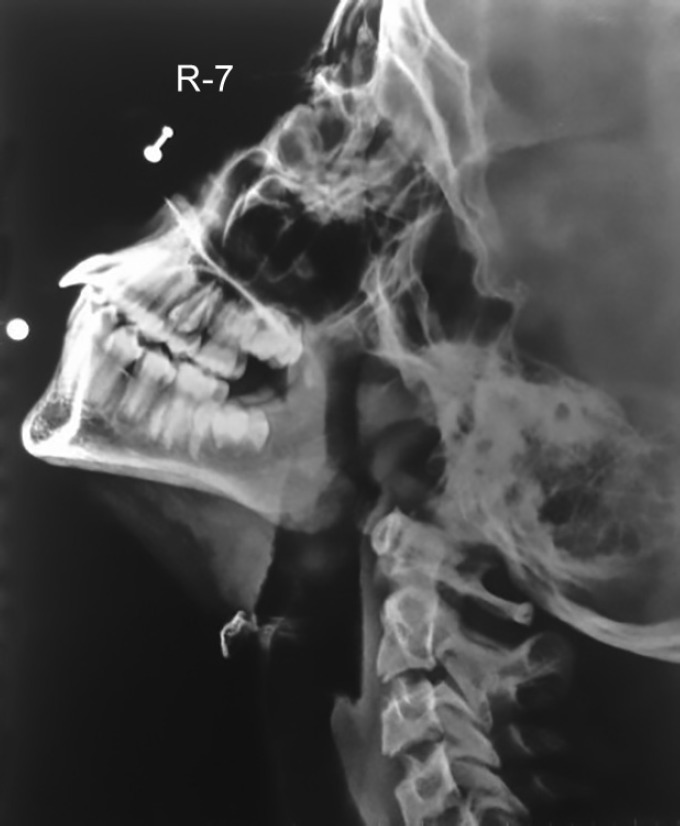
Lateral cephalometric radiograph of the nasopharynx showing enlargement of the adenoid gland which causes narrowing of the air passage

The oral examination also showed the presence of a red bright erythematous tongue(strawberry tongue) with tongue tie (Fig. 2), dry erythematous fissured lips that bled easily, along with prognathic maxilla and protruded maxillary teeth (Fig. 3). Differential diagnosis of recurrent Kawasaki disease and scarlet fever was made because of the presence of strawberry tongue. As the patient was not having a history of sore throat or tonsillar exudate, the chances of scarlet fever were minimal. No oral exudates, ulcerations, or Koplik's spots were reported. The family history did not reveal any similar complaints in the family. The child was diagnosed with Kawasaki disease at 4 years of age. Lateral cephalometric radiograph of the nasopharynx showed enlargement of adenoid gland causing narrowing of the air passage (Fig. 4).

**Fig. 5:**
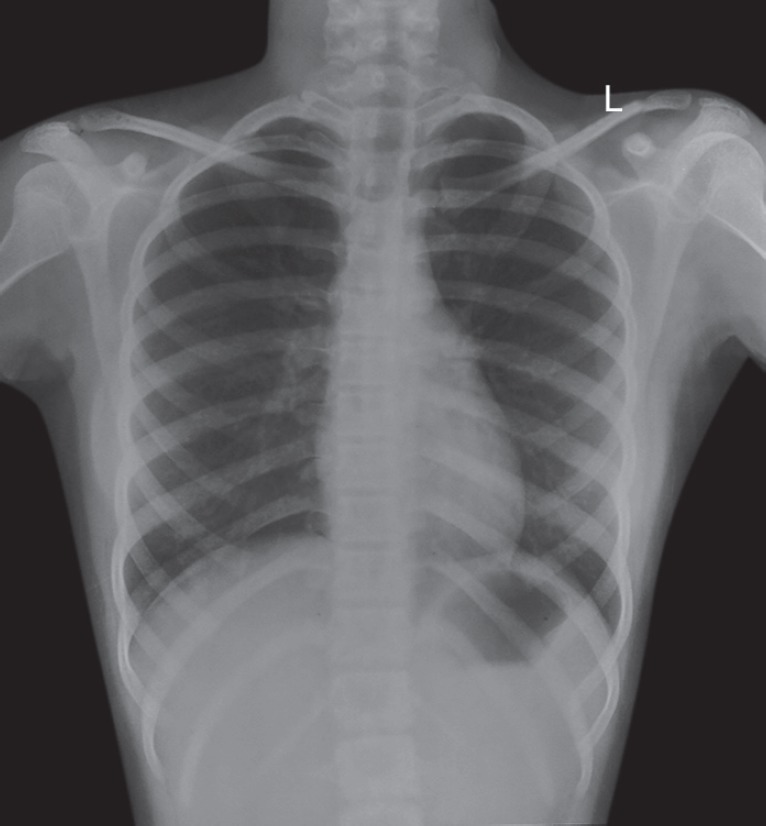
X-ray of the chest showing an enlarged heart, suggestive of cardiac involvement

The patient was referred by us to a pediatrician for further checkups as there was suspicion of recurrence of Kawasaki disease. Investigations showed total white blood cells (WBC) count of 15,600/mm^3^ with 70% neutrophils, increased ESR (60 mm at the end of 1 hour) and serum C-reactive protein (96 mg/L) and low platelet count (80,000/mm^3^). Urine examination and culture study showed sterile pyuria. Tuberculosis test was negative. Electrocardiogram (ECG) reports showed cardiac changes. X-ray of the chest showed an enlarged heart and cardiac involvement (Fig. 5).

Generalized lymphadenopathy along with desquamation of the fingertips of the girl was seen within 2 days of reporting. Based on the investigations report and clinical picture, a diagnosis of recurrent Kawasaki disease was made by the pediatrician. She was treated with a single dose of 2 g/kg of intravenous immunoglobulin, and regular daily aspirin was adviced. The discharge was given after 3 days and the patient was free of the oral signs and symptoms within a month.

## DISCUSSION

Kawasaki disease is predominantly found in children under the age of 5 years but in some case, it has been reported in adults too.^3,6,7^ The recurrence rate is high in patients who have a previous history of Kawasaki disease and present with clinical signs consistent with recurrence.

Vasculitis is the primary pathological process in Kawasaki disease. The acute phase is characterized by the inflammation of all the arteries of the pericardium, myocardium and endocardium.^8^ Sudden death may occur in the acute phase of the illness in 4% cases of untreated Kawasaki disease because of aneurysmal thrombus formation, myocardial infarction or dysrhythmia. For one percent of cases involving the heart valves, antibiotic prophylaxis is needed prior to relevant dental procedures to prevent infective endocarditis.^10^

Kawasaki disease etiology is idiopathic in origin. Bacterial superantigen toxin is known to be the recent etiological agent of the disease. The incidence and severity of aneurysm formation are reduced significantly by giving a single dose of intravenous immunoglobulin (2 g/kg) These immunoglobulins also provide remarkable symptomatic relief to the patient if given as soon as possible after diagnosis. Low dose aspirin and antipyretics can also be used although their efficacy remains unproven.^9^

Recurrence rates of this disease vary from 3% in Japan to 0.8% in the United States. As the age advances, the recurrence rate of the disease increases, while maximum recurrences will occur during the 2 years of the initial attack. Multiple recurrences are rarely seen in almost 2% of the patients suffering from this disease. The patients who had previous treatment with immunoglobulin have been identified as the only factor which predisposes to recurrence, as seen in our case report.^11^

The disease has high mortality and morbidity rate if not treated early, hence an early diagnosis and treatment are important in managing this condition. The oral findings are a characteristic feature of this serious disease hence many cases might first report to the dental clinician only. As we can see in this case report that it was the dentist's role in identifying the disease by history and clinical features, and referring it to the pediatrician for confirming the recurrence of Kawasaki disease by various tests. Thus the timely diagnosis of the disease prevented mortality in this patient. So, the dentists should be cautious and alert in doing patients with a history of Kawasaki disease, because there is a possibility of recurrence of the disease and also as these patients have valvular heart defects, they might frequently require prophylactic antibiotic treatment before the needed dental procedure.

Till date very rarely the occurrence of Kawasaki disease with lip swelling has been reported in the literature.^13^ Only one study done by Faden has shown the association of Kawasaki disease with lip swelling and that is also the upper lip. We present a very rare case report of recurrent Kawasaki disease with lower lip swelling as an oral manifestation of KD, which is a rare occurrence and not reported before.

There seems to be less awareness of this disease among the dentists, hence this condition goes unnoticed leading to few citations of this disease in the dental literature.

## CLINICAL SIGNIFICANCE

It is necessary to create awareness of Kawasaki disease in the dental community as it is a rare disease and some cases might go unnoticed due to lack of knowledge of the treating dentist. Thus the proper knowledge helps in the timely diagnosis of the disease and decreases the mortality rate in these patients. Children who show the oral features of this disease should be treated seriously by the dentist.
